# Targeted Research to Improve Invasive Species Management: Yellow Crazy Ant *Anoplolepis gracilipes* in Samoa

**DOI:** 10.1371/journal.pone.0095301

**Published:** 2014-04-15

**Authors:** Benjamin D. Hoffmann, Saronna Auina, Margaret C. Stanley

**Affiliations:** 1 CSIRO Ecosystem Sciences, Tropical Ecosystems Research Centre, Darwin, NT, Australia; 2 Centre for Biodiversity and Biosecurity, School of Biological Sciences, The University of Auckland, Auckland, New Zealand; Stanford University, United States of America

## Abstract

Lack of biological knowledge of invasive species is recognised as a major factor contributing to eradication failure. Management needs to be informed by a site-specific understanding of the invasion system. Here, we describe targeted research designed to inform the potential eradication of the invasive yellow crazy ant *Anoplolepis gracilipes* on Nu’utele island, Samoa. First, we assessed the ant’s impacts on invertebrate biodiversity by comparing invertebrate communities between infested and uninfested sites. Second, we investigated the timing of production of sexuals and seasonal variation of worker abundance and nest density. Third, we investigated whether an association existed between *A. gracilipes* and carbohydrate sources. Within the infested area there were few other ants larger than *A. gracilipes*, as well as fewer spiders and crabs, indicating that *A. gracilipes* is indeed a significant conservation concern. The timing of male reproduction appears to be consistent with places elsewhere in the world, but queen reproduction was outside of the known reproductive period for this species in the region, indicating that the timing of treatment regimes used elsewhere are not appropriate for Samoa. Worker abundance and nest density were among the highest recorded in the world, being greater in May than in October. These abundance and nest density data form baselines for quantifying treatment efficacy and set sampling densities for post-treatment assessments. The number of plants and insects capable of providing a carbohydrate supply to ants were greatest where *A. gracilipes* was present, but it is not clear if this association is causal. Regardless, indirectly controlling ant abundance by controlling carbohydrate supply appears to be promising avenue for research. The type of targeted, site-specific research such as that described here should be an integral part of any eradication program for invasive species to design knowledge-based treatment protocols and determine assessment benchmarks to achieve eradication.

## Introduction

Despite growing global efforts to curtail biological invasions, the spread of invasive alien species remains an increasing management problem [1], [2], [3]. Although eradications of some taxa are now becoming routine [4], this is not so for most biota [5], [6], with the lack of knowledge of the biology of the target species being recognized as a major contributing factor for failure [7], [8]. Such biological information is vitally important for at least two reasons. First, it is an essential component of risk analysis, assessing the risks posed by an invader, the risks associated with management, and the likelihood of success of management actions [9], [10]. Second, specific biological knowledge is often vital for the development of effective management protocols [7]. It is often important that this knowledge is gained on-site because the biology and ecology of species can vary greatly between the native and exotic range, as well as among exotic ranges [11], [12], [13]. For example, the northern tamarisk beetle *Diorhabda carinulata*, introduced into North America as a biocontrol agent against *Tamarix* spp. was effective in some regions, on some species, but failed in others. Subsequent research found different *Tamarix* species and genotypes yielded different responses by the beetle [14], and that over-wintering adults could not survive below the 38^th^ parallel [15]. Determination of the mismatches between host and control agent and the agent and climatic suitability has led to more targeted use of this beetle and to better management outcomes.

Many ant species that have been accidentally spread throughout the world have significant economic, environmental and social impacts in areas that they now infest [16], [17], [18]. Although there have been many attempts at eradicating exotic ant incursions, few efforts have been successful, and a lack of specific biological knowledge is believed to have been a major contributing factor [6], [8], [19]. For example, baiting during periods when queen brood are in pupal stage will not achieve eradication because these pupae will not be affected by the treatments and will emerge to initiate new colonies. A lack of site-specific information can also hinder effective assessment of treatment success. For example, reduced activity following treatment may simply reflect a normal activity cycle rather than a treatment effect. Clearly, if management decisions and protocols based on the target species’ biology are to be effectively applied, the biological knowledge is therefore best obtained on-site.

**Figure 1:**
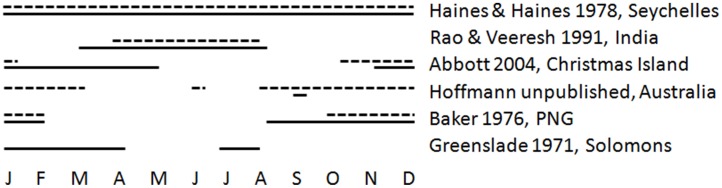
Annual production of *Anoplolepis gracilipes* sexual brood reported in the literature. Data are ordered from the Seychelles in the Indian Ocean to the Solomon Islands in the Pacific Ocean. In all instances, samples were collected year-round. Solid lines indicate the presence of queen brood and dashed lines indicate male brood.

**Figure 2:**
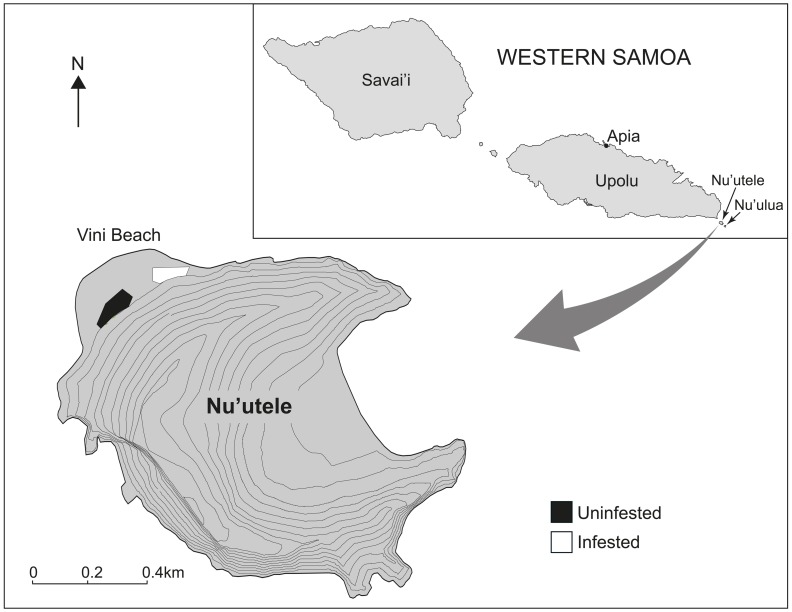
Nu’utele Island of the Aleipata Island group of Western Samoa, and the location of the two study areas. One of the study areas is infested with *Anoplolepis gracilipes* (white polygon) and the other is uninfested (black polygon).

One of the most notable invasive ants is the yellow crazy ant, *Anoplolepis gracilipes*. This species has a pan-tropical distribution [20], and is well known to have great variation in its abundance [21], [22], [23], [24], impacts [25], [26] and reproductive phenology [27], [28], [29] (Figure 1). Yellow crazy ant has invaded Samoa’s Aleipata islands (Figure 2), which are considered to be of great regional conservation significance because they are uninhabited, relatively pristine, contain many species threatened throughout greater Samoa, and lack many exotic species present within greater Samoa. The presence of *A. gracilipes* on these islands is therefore of significant conservation concern.

Prior work on the Aleipata islands has shown that *A. gracilipes* is well-distributed over the island of Nu’ulua [30], but has a much more restricted distribution on the island of Nu’utele [31]. Because of its restricted distribution, the prospect of eradicating *A. gracilipes* from Nu’utele was recently investigated [32]. Here we describe research on the biology of *A. gracilipes* on Nu’utele that was designed to underpin a potential eradication program. First, we determine whether resident invertebrates are affected by *A. gracilipes*. An understanding of the impacts of the invasive species is required for informing a comprehensive risk analysis to determine if eradication should be considered. Second, we assess the timing of production of sexuals, and compare seasonal variation of nest density and abundance of *A. gracilipes*. Information on the timing of production of sexuals, and seasonal variation of worker abundance and nest density is required for the strategic design of treatments and post-treatment monitoring. Finally, we investigate associations between *A. gracilipes* and carbohydrate sources. Carbohydrate supply is increasingly recognised as a strong driver of ant invasions [12], [33], [34], [35], so controlling carbohydrate supply shows promise as an indirect control method for invasive ants. From its reported biology from other exotic locations throughout the world, particularly within the Australasian-Pacific region, we expected that: 1) *A. gracilipes* would reduce species richness and abundance of other invertebrates, particularly ants; 2) reproduction of sexuals would occur between approximately September and November; 3) greatest population levels and nest densities would occur during the tropical wet season (November to April); and 4) it would be highly associated with carbohydrate sources.

## Methods

### Study Sites and Sampling Periods

Nu’utele island (14° 03′ 50″S 171° 25′ 25″E) is the remains of volcanic tuff cone. The substrate of the sampling areas was coral debris overlain with sandy peat, and the vegetation was littoral forest with the overstorey dominated by numerous tree species, including T*erminalia catappa* and *Barringtonia asiatica*, and the mid-storey was dominated by *Macaranga harveyana, Morinda citrifolia* and *Hibiscus tiliaceus*.

A permit to conduct sampling was provided by the Samoan Ministry of Natural Resources and Environment. The field studies did not involve endangered or protected species. Nothing is known of the invasion history of *A. gracilipes* on Nu’utele, but it is believed to have arrived only within the last decade [32]. At the time of this study, there was only one *A. gracilipes* population large enough to conduct detailed research within, being at Vini beach, but two other smaller populations were present on the other side of the island. Work was conducted throughout the accessible area of the Vini beach infestation (Figure 2) and a nearby uninfested area. These areas (hereafter referred to as sites) were paired as far as practicable by: (1) elevation, being near the base of the steep incline; and (2) vegetation structure having an interlocking canopy and a dense understory. The vegetation structure and composition of these sites appeared comparable to most vegetation covering the island.

This design is inherently pseudoreplicated [36] because the treatment (infestation) is not replicated, but this was unavoidable. However, within comparative mensurative experiments (as opposed to manipulative experiments) such as this, pseudoreplication is more an issue about whether samples from a single ‘treatment’ are collected within a restricted range of the possible area, or from throughout the greatest range of space possible [36]. Therefore all work was conducted throughout as great an area as possible within the infested site, up to approximately 20 m of the infestation boundary to exclude edge effects, and throughout a comparable area in the uninfested site. Additionally we lowered the significance value of statistical tests to P≤0.025 so that only very large differences are given recognition.

Two field trips were conducted to obtain multiple samples, the first in October 2010 and the second in May 2011. Although monthly data would have been preferable, logistical constraints did not allow more than two trips, so these dates were chosen because these months approximate the extremes within the *A. gracilipes* abundance and sexual reproduction cycles throughout the Australasian-Pacific region [27], [28]. It was anticipated that Samoan populations of *A. gracilipes* would have similar dynamics, with reproduction of sexuals and lowest worker abundance in October, and no reproduction of sexuals coupled with greater worker abundance in May.

The boundaries of the infestation were delimited on both sample times using visual assessments of the presence/absence of *A. gracilipes* workers. The assessments were conducted by a team of people spaced 5 m apart walking in parallel. Assessments consisted of an approximately four second search for *A. gracilipes* on the vegetation and substrate. Assessments were conducted haphazardly but regularly (approximately one per every 2 m). This mapping technique is used extensively in Australian *A. gracilipes* management programs (B. Hoffmann, unpublished data). Where the infestation boundary was capable of expansion (ie not along a beachfront), a slight (<20 m) expansion was found between the two sample times, but the area surveyed in May 2011 was the same as for October 2010.

### Impacts

The impacts of *A. gracilipes* on the abundance of epigeic and arboreal invertebrates were assessed using pitfall traps and foliage beats respectively. Pitfall traps were plastic containers with an internal diameter of 65 mm, one third filled with ethylene glycol as a preservative. Traps were used in 20 plots throughout the infested site and another 20 plots throughout the uninfested site. Plots comprised of three traps set in triangle formation, spaced 2 m apart and operated for 48 hours. Plots were spaced at least 10 m apart to maximise independence. The same plot locations were used for the two sample months. All macroinvertebrates >1 mm in length were identified to ordinal level, except ants, which were identified to species level following [37]. Voucher specimens of the ant species were placed in the CSIRO Darwin ant collection. Pitfall trap data were pooled for each plot.

Twelve foliage beat samples were collected along a single transect within each of the infested and uninfested sites. Where possible, assessments were made every 4 m along the transect from the closest plant >2 m high, or low lying branch of a tree. If a unique sample could not be made at a subsequent sample point (e.g. the closest plant was a tree with no low lying branches), the sample was conducted at the next 4 m location, and thus the transect was extended as far as needed to collect 12 samples in each site. The selected foliage was beaten four times over a 1×1 m white canvas, and all invertebrates that fell onto the canvas were collected. The transect locations were approximately the same for the two sample months.

Most non-ant invertebrates from most orders collected in both pitfall traps and foliage beats were capable of flight and thus were likely to be highly mobile. Because of this issue, coupled with the relatively small size of the infested site, it was deemed that analyses of individual groups would not be sufficiently credible due to the high likelihood of continual incursion of individuals from outside the infested area. Spiders (Arachnida), however, are relatively sedentary, and are well known to be sensitive to exotic ant invasions [38], [39], so ordinal-level analyses were restricted to this group.

The potential impact of *A. gracilipes* on hermit crabs (*Coenobita* spp.) was assessed by counting the number of crabs found within one minute in 10 transects (5×1 m) in both the infested and uninfested site during the early evening between 7 and 9 pm. Transects were established haphazardly throughout the sites, and were positioned at least 10 m apart. Different locations were used for the two sample months. Crabs were divided into two arbitrary size classes: small (<5 mm across the carapace) and large (>5 mm across the carapace) on the basis that we anticipated impacts on crabs to be size dependent.

### Reproductive Phenology

The reproductive phenology of *A. gracilipes* was assessed by quantifying male and queen pupae production. All pupae were collected from within ten nests haphazardly located throughout the site, and subsequently identified as being either a worker, male or queen in the laboratory. Different nests were excavated during the two sample months.

### Seasonal Variation of Abundance

Seasonal variation in the abundance of *A. gracilipes* workers was measured indirectly from pupae counts collected as part of measurements to determine the reproductive phenology, and directly from worker counts on cards and at fish lures. Card and lure counts were conducted at the same sample points along transects on the same day, with the card assessments being conducted prior to fish lure assessments. Eleven sample points were spaced 5 m apart along each of four parallel 50 m transects spaced 5 m apart. The same sample points were used for the two sample months.

Cards were 20 cm×20 cm laminated paper divided by pen into four 10 cm×10 cm squares. At each assessment point a card was placed on the ground with the edges in contact with substrate as far as possible to allow easy access for the ants to walk onto the card. The card was observed for 20 seconds, and the first square accessed by an *A. gracilipes* worker was the only square used for the assessment. The number of *A. gracilipes* workers that walked over that square was counted over the following 30 seconds. If no ant walked over the grid in the first 20 second assessment period, then the square to be used was determined by the first ant that walked over the grid in the 30 second assessment period. The abundance counts were pooled for each transect, then averaged across transects.

Fish lures were a teaspoon of canned tuna placed directly onto the ground. *Anoplolepis gracilipes* abundance at each lure was scored after 20 minutes according to the following scale: 0 =  no ants; 1 = 1 ant; 2 = 2–5 ants; 3 = 6–10 ants; 4 = 11–20 ants; 5 = 21–50 ants; 6 = 50–100 ants; and 7 = >100 ants. The scaled abundance measures were averaged for each transect, then averaged among transects.

### Seasonal Variation of Nest Density

Seasonal variation of nest density was quantified in four 5×5 m plots haphazardly located throughout the extent of the infested area, with plots always being >20 m apart. Within each plot, nests were located by disturbing all leaf litter and surface materials. Nests were defined as locations from where ants were emerging (i.e. a hole in the ground), or where pupae were aggregated. Nests<50 cm apart were considered to be the same nest because subterranean nest entrances located closer than 50 cm apart are predominantly joined to a single nest chamber (B. Hoffmann, pers. obs.).

### Carbohydrate Supply

Honeydew-producing insects and plants with extra-floral nectaries (EFNs) or with nectar sources (e.g. fruit exuding liquid that was tended by ants) were quantified in the May sample in both sites every 2 m along the same transects used for foliage beats to assess *A. gracilipes* impacts. At each sample location the closest plant >2 m high was identified, and the presence/absence of honeydew-producing insects, EFNs, and nectar sources, as well as any interaction with *A. gracilipes* were noted. The abundance of honeydew-producing insects was noted as being either an individual, few (2–10 individuals), or a cluster of >20 individuals.

### Analyses

The non-parametric Mann-Whitney U-Test was used to compare data from infested and uninfested plots, and the Wilcoxon matched pairs T-test was used to compare data from infested samples only. The occurrence of honeydew-producing insects and plants with EFNs along the carbohydrate supply transects were compared between the infested and uninfested plots using 2-tailed Chi-square tests.

## Results

### Impacts

#### Ants in pitfall traps

A total of 24 ant species from 15 genera were collected within pitfall traps over both sampling times; 18 species from 13 genera within the October sample and 20 species from 13 genera within the May sample. The most abundant species (excluding *A. gracilipes*) were *Pheidole umbonata* (46.2% of total abundance of all species excluding *A. gracilipes* within both sample months), the exotic tramp *Paratrechina longicornis* (18.6%), *Odontomachus simillimus* (17.4%) and another exotic tramp, *Tetramorium bicarinatum* (7.2%). The relative contribution of these four species was very similar between the two sample months.

**Figure 3:**
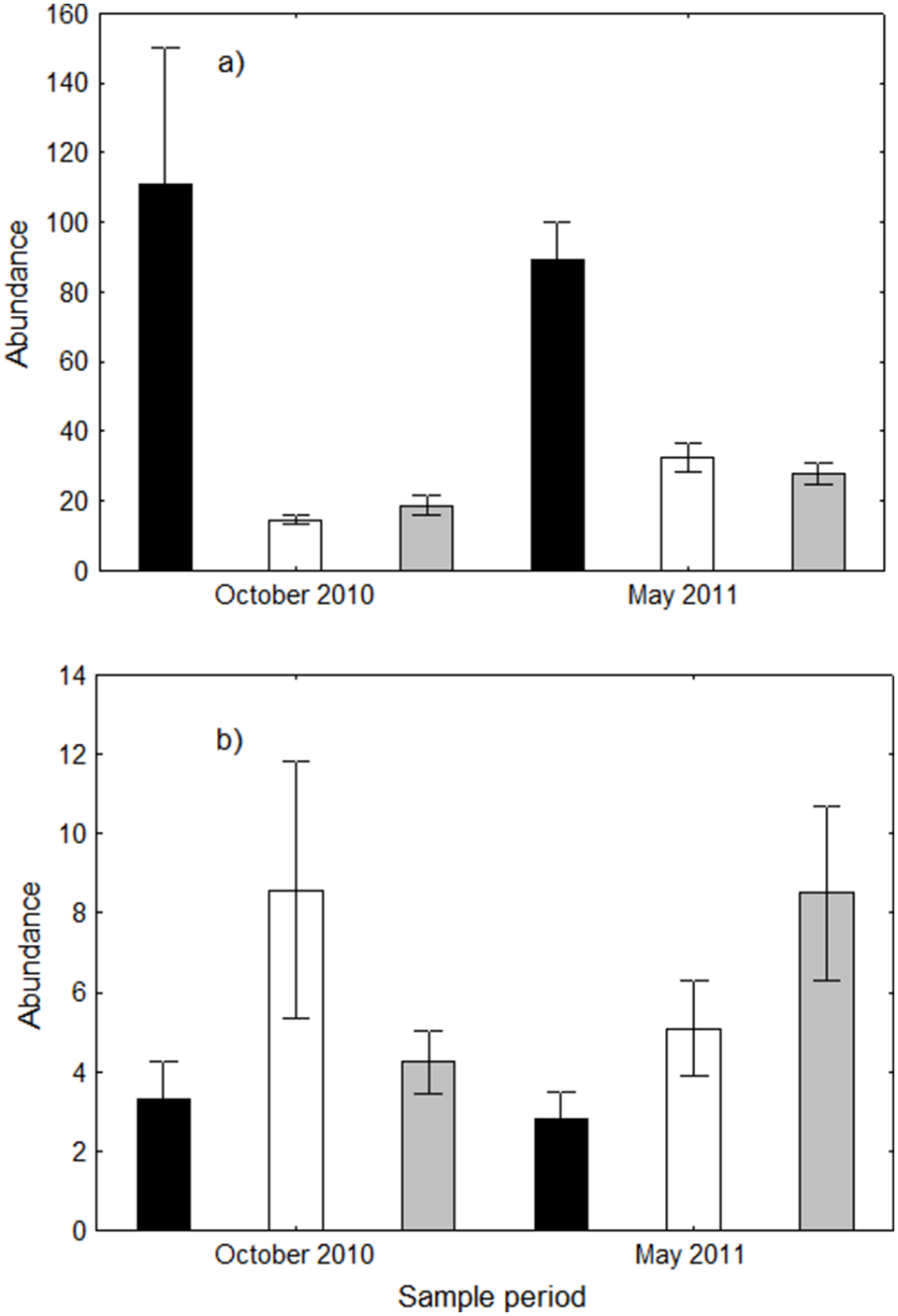
*Anoplolepis gracilipes* abundance compared to other ant abundance. Data are mean (± SE) abundance within plots for *Anoplolepis gracilipes* (black bars) and the abundance of all other ants within the infested site (white bars) and uninfested site (grey bars) within a) pitfall traps, and b) foliage beats during the October 2010 and May 2011 sampling periods.

*Anoplolepis gracilipes* abundance within pitfall traps at the infested site was always significantly greater than the abundance of all other ants combined (Tables 1, 2), being 7.6 and 5.9 times greater than other ant abundance within the infested and uninfested sites respectively in the October sample, and 2.7 and 3.5 times greater respectively in the May sample (Figure 3a). We found *A. gracilipes* abundance within pitfall traps was lower within the May sample, not greater as found by card counts and tuna lures, but this is solely due to an exceptionally large number of *A. gracilipes* (n = 815) falling into a single trap within the October sample, presumably because the trap was placed directly beside a nest.

**Table 1:** Results of Wilcoxon matched pairs T-tests for comparisons of *A. gracilipes* abundance vs native ant abundance within pitfall traps in infested plots, for the two sample periods.

Sample period	*A. gracilipes* abundance	Other ant abundance	T	z	P
October 2010 sample	111±39	14.7±1.3	0	3.92	**<0.0001**
May 2011 sample	89±11	33±4	0	3.72	**<0.0002**

Bold indicates significance of P≤0.025. Abundance data are mean ± SE.

**Table 2:** Results of Mann-Whitney U-tests of ant pitfall trap and foliage beat data between infested and uninfested plots for the two sample periods.

Metrics	Pitfall traps					Foliage beats				
	Infested	Uninfested	U	z	P	Infested	Uninfested	U	z	P
*October 2010 sample*										
*A. gracilipes* abundance in infested site vs other ant abundance in uninfested site	111±39	19±2.9	27	4.667	**<0.0001**	3.3±0.9	4.3±0.8	59	−0.722	0.466
Non-*A. gracilipes* ant abundance	14.7±1.3	19±2.9	175.5	−0.649	0.516	8.6±3.3	4.3±0.8	64	0.433	0.665
Ant species richness excluding *A. gracilipes*	3.7±0.4	2.3±0.2	166	0.906	0.365	2.3±0.5	1.5±0.2	51	1.184	0.237
Non-*A. gracilipes* ant abundance excluding *Pheidole umbonata*	5.3±1.1	11.4±1.8	92	−2.91	**0.0035**					
*May 2011 sample*										
*A. gracilipes* abundance vs other ant abundance	89.2±10.8	27.7±3.1	35.5	4.21	**<0.0001**	2.8±0.7	8.5±2.2	31	−2.338	**0.019**
Non-*A. gracilipes* ant abundance	32.7±4.1	27.7±3.1	157	0.658	0.511	5.1±1.2	8.5±2.2	54.5	−0.981	0.326
Ant species richness excluding *A. gracilipes*	5.9±0.4	2.9±0.2	27.5	4.444	**<0.0001**	1.9±0.4	2.4±0.3	54.5	−0.981	0.326
Non-*A. gracilipes* ant abundance excluding *Pheidole umbonata*	15.2±3.6	19.2±2.6	124	−1.623	0.105					

Bold indicates significance of P≤0.025. Data are mean ± SE.

Other ant abundance within pitfall traps was not statistically different between infested and uninfested plots in both sample months (Figure 3a, Table 2). However, other ant abundance was dominated by a single species, *P. umbonata* (51% and 44% in the October and May samples respectively), and with this species excluded from analysis, other ant abundance was significantly lower within the infested plots (average 5 ants per plot) compared to the uninfested plots (11 ants per plot) within the October sample, and lower (15 vs 19 ants), albeit not significantly, in the May sample (Table 2). This lack of significance in the May sample is predominantly attributable to a very high number of *T. bicarinatum* (48 ants) caught within a single trap, presumably placed beside a nest, but even with this trap removed, the difference between the two sites remained statistically insignificant (Mann-Whitney U-test, P = 0.08).

**Figure 4:**
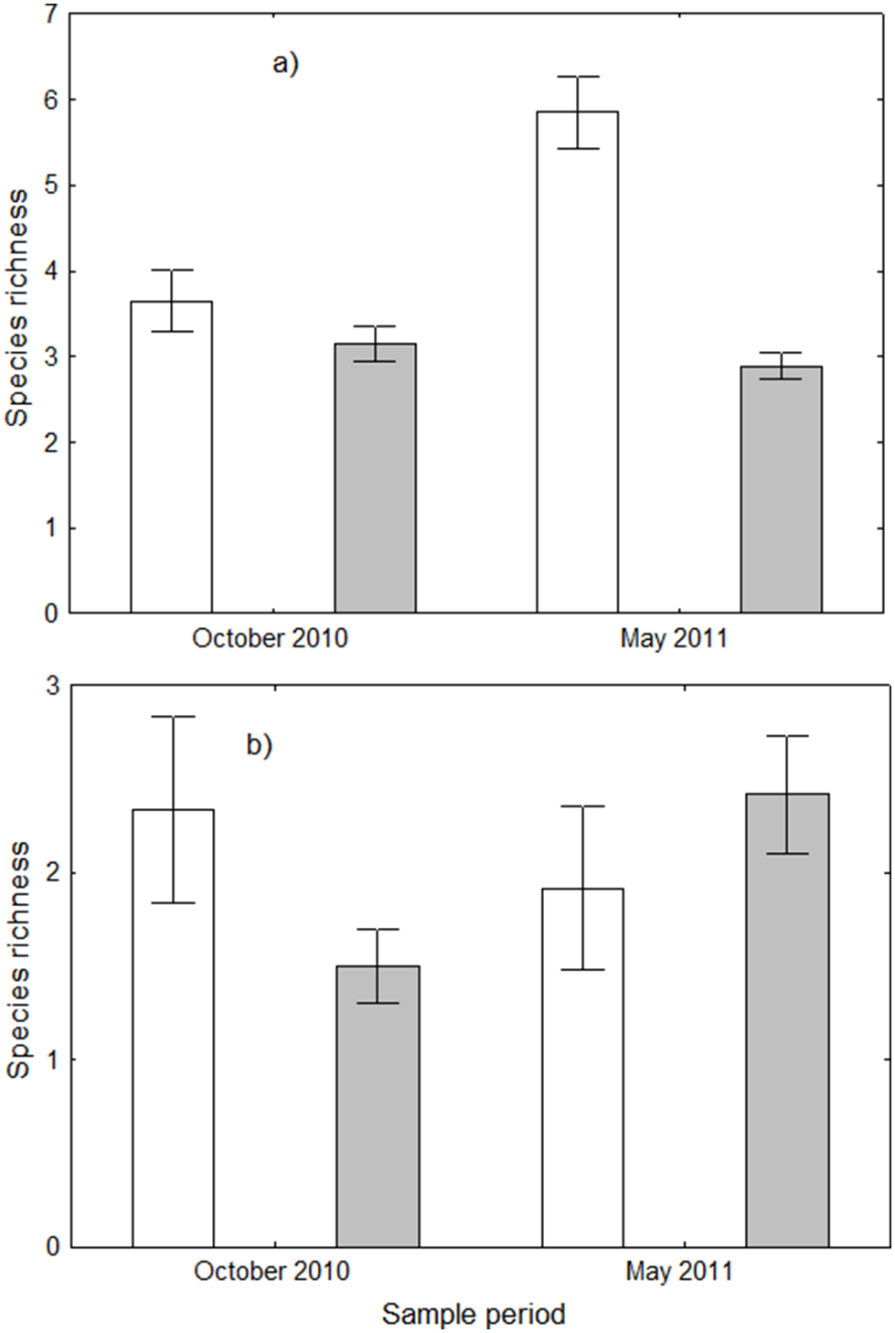
Ant species richness. Data are mean (± SE) ant species richness, excluding *Anoplolepis gracilipes*, within plots in the infested (white bars) and uninfested site (grey bars) within a) pitfall traps, and b) foliage beats during the October 2010 and May 2011 sampling periods.

Ant species richness per plot within pitfall traps, excluding *A. gracilipes*, was always greater within the infested site (Figure 4a, Table 2), having an average of six species per plot in the infested site vs three in the uninfested site. A total of 14 species were found within the infested site and 11 in the uninfested site in the October sample, and 18 vs 7 in the May sample.

#### Ants in foliage beats

Nine ant species from seven genera were collected within foliage beats over both sample times, with the October and May samples each having seven species. Excluding *A. gracilipes*, four exotic tramp species comprised 86% of total abundance of ants in foliage beats within both samples combined, being *Tapinoma melanocephalum* (39%), *P. longicornis* (23%), *Monomorium floricola* (19%) and *T. bicarinatum* (5%). The contribution of these species within the two sample months varied greatly, with that of *P. longicornis* being 34% and 9% in the October and May samples respectively, 31% and 6% respectively for *M. floricola*, 28% and 52% for *T. melanocephalum*, and 0% and 12% for *T. bicarinatum*.

Within the infested site the abundance of other ants in foliage beats was 2.6 and 1.8 times greater than that of *A. gracilipes* in the October and May samples respectively, (Figure 3b), but these differences were not statistically significant (Wilcoxon matched pairs T-test, T = 14.5, z = 1.325, P = 0.185 for October and T = 17, z = 1.423, P = 0.155 for May) due to large variation among the samples. Similarly, other ant abundance in foliage beats within the uninfested plots was not statistically different from *A. gracilipes* abundance in the October sample, but was statistically greater in the May sample (Figure 3b, Table 2). There was no significant difference between the abundance or species richness of other ants in foliage beats between the infested and uninfested plots in either sample months (Figures 3b, 4b; Table 2).

#### Other macro-invertebrates in pitfall traps

**Figure 5:**
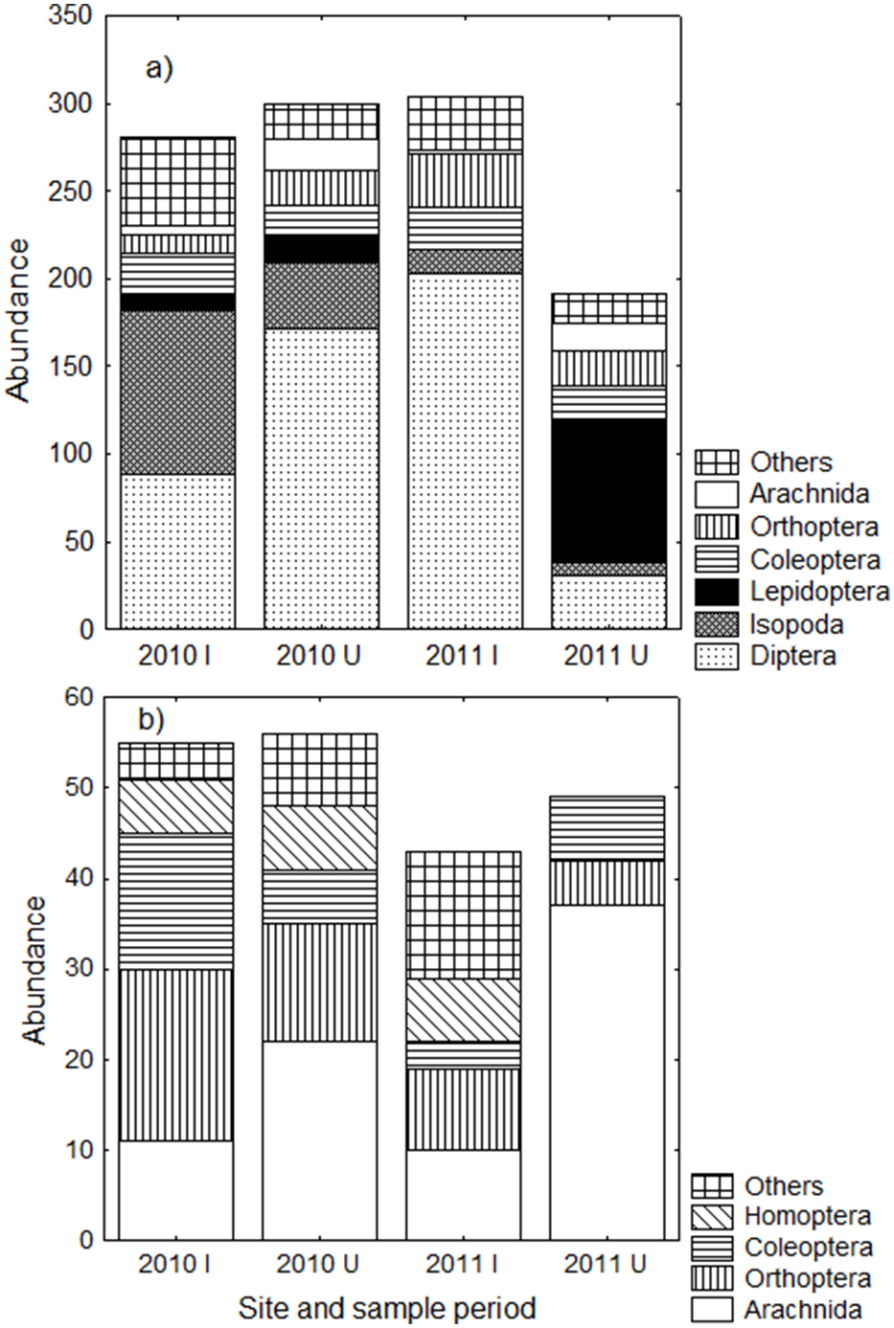
Abundance of the other macro-invertebrate orders. Data are total abundance of non-ant invertebrates collected in a) pitfall traps and b) foliage beats within sites infested (I) or uninfested (U) with *Anoplolepis gracilipes* during the October 2010 and May 2011 sampling periods.

Other macro-invertebrates from 11 orders were collected in pitfall traps. Flies were the predominate group collected (46% of all samples combined), followed by isopods (14%), moths and butterflies (8%) and crickets (7%) (Figure 5a). There was no difference in the overall abundance or ordinal richness of other macro-invertebrates in pitfall traps between the infested and uninfested sites for either of the two sample months (Figure 5a, Table 3). There was a clear trend of fewer spiders within the infested site (5 vs 18 individuals in October and 2 vs 16 in May), however, this was not statistically significant (Table 3), presumably because so few individuals were collected, resulting in many tied ranks in the statistical test.

**Table 3:** Results of Mann-Whitney U-tests of other macro-invertebrate data from pitfall traps and foliage beats between infested and uninfested plots in the two sample periods.

Metrics	Pitfall traps					Foliage beats				
	Infested	Uninfested	U	z	P	Infested	Uninfested	U	z	P
total abundance	14.1±1.9	15±2.2	196	−0.09	0.924	4.6±0.7	4.6±1	63.5	0.462	0.644
ordinal richness	5.1±0.4	4.4±0.4	164	0.96	0.337	2.6±0.4	2.8±0.3	63.5	−0.462	0.644
Spider abundance	0.3±0.1	0.9±0.2	16.5	−0.653	0.514	0.9±0.4	1.8±0.6	55.5	−0.924	0.356
*May 2011 sample*										
total abundance	15.2±3.9	10.6±4.3	109	2.06	0.039	3.6±0.6	5.3±0.8	48	−1.357	0.175
ordinal richness	3.9±0.4	3.8±0.4	178	−0.03	0.977	2.1±0.3	2.3±0.25	64.5	−0.404	0.686
Spider abundance	0.1±0.1	0.9±0.2	8	−0.639	0.523	0.8±0.5	3.1±0.8	30	−2.397	**0.017**

Data are mean ± SE. Bold indicates significance of P≤0.025. Data are mean ± SE.

#### Other macro-invertebrates in foliage beats

Other macro-invertebrates from eight orders were collected in foliage beats. Spiders were the predominate group collected (37% of all samples combined), followed by crickets (21%), and beetles (14%) (Figure 5b). Just as for other macro-invertebrate data from pitfall traps, there was no difference in overall abundance or ordinal richness between the infested and uninfested sites within either of the two sample months (Table 3). There were fewer spiders within the infested site in both sample months, significantly so in the May sample (Table 3).

#### Hermit crab counts

In the October sample, when *A. gracilipes* abundance was lowest, the infested site had significantly fewer (approximately one quarter) of the crabs per plot (average = 1.3±0.4) of the uninfested site (average = 5.3±1.6; Mann-Whitney U Test: U = 104, Z = −2.5, P = 0.0098). This result was primarily driven by the prevalence of large crabs (Mann-Whitney U test: U = 98.5, Z = −2.73, P = 0.0063) as there were too few small crabs collected to produce a statistical difference in this size class (Mann-Whitney U test: U = 179.5, Z = −0.54, P = 0.5885). Only seven small crabs were found in the infested site compared to 28 in the uninfested site, but the proportion of small crabs to the total count was consistent between the two sites (27% and 26% respectively), indicating that any factor affecting hermit crab abundance applied equally to both size classes.

The difference in crab abundance between the infested and uninfested sites were even more pronounced in the May sample when *A. gracilipes* abundance was greater, with only four large crabs being found in the infested site, compared to 54 crabs of both size classes combined (an average of 2.7 crabs per transect) in the uninfested site (Mann-Whitney U Test: U = 34, Z = −4.477, P<0.0001).

### Reproductive Phenology

Male pupae and larvae were found in all nests in October, with their combined contribution to the brood averaging 22.8% and ranging between 4.2–46.8%. Male brood were found in only one nest in May, comprising 20% of the brood. Queen pupae were found only in May within a nest that was not part of this formal assessment.

### Seasonal Variation of Abundance

Worker abundance from card counts averaged 30±4 ants in October and 83±6 in May. The average abundance score from tuna lures was 4 (being between 11–20 ants) in October and 6.7 (>100 ants) in May.

### Seasonal Variation of Nest Density

Nest density was greater in May when population levels were also greater. The four plots from the October sample contained 6, 2, 5 and 6 nests respectively. The plot containing only two nests is considered to be atypical as it was within a stand of *Pisonia grandis*, which is known to be unfavourable for invasive ants [40], [41]. Therefore, excluding this plot, the average nest density was one per 4.4 m^2^. In the May sample, the nest density was approximately double of that in October, with the four plots containing 17, 12, 10 and 7 nests respectively, equating to an average nest density of one per 2.2 m^2^.

### Carbohydrate Supply

Multiple unidentified species of scale and at least one mealy bug species were found on six tree species (Table 4). The only interaction noticed between *A. gracilipes* and these insects was with scales on Indian mulberry *Morinda citrifolia* and mealy bugs on coconut, *Cocos nucifera*, but all of the insect species were found within the infested area. Six plant species were found to have extrafloral nectaries on their leaves or carbohydrate sources accessible to ants (Table 4), but *A. gracilipes* was found attending these sources only on the Indian mulberry *Morinda citrifolia* and beach hibiscus *Hibiscus tiliaceus*.

**Table 4:** Non-floral carbohydrate sources (plants with extra floral nectar (EFN) sources and honeydew-producing insects) present on Nu’utele, and records of interaction between *Anoplolepis gracilipes* and these carbohydrate sources.

Commonname	Scientificname	Description	A. *gracilipes* interaction observed
**Plants**			
Indian Mulberry	*Morinda citrifolia*	Nectar supply at floral inserts on fruit	Yes
Passionfruit	*Passiflora foetida*	EFN location unclear, but *Passiflora*known to have EFN	No
Passionfruit	*Passiflora sp.*	EFN location unclear, but *Passiflora*known to have EFN	No
Tropical almond	*Term* *inalia* *catappa*	EFN pair at base of leaf	No
	*Macaranga* *harveyana*	EFN at base of leaf	No
Beachhibiscus	*Hibiscus tiliaceus*	EFNs at base of leaf	Yes
**Insects**			
Mealy bug		Found on *Barringtonia asiatica*,*Cocos nucifera*, *Mikanika micrantha* and*Omalanthus nutans*,	Yes, but only mealy bugs on*Cocos nucifera* were within theinfested site
Scale insects		Found on *Barringtonia asiatica*, *Macaranga* *harveyana* and *Morinda citrifolia*	Yes on *Morinda citrifolia*,no for all others. Scale insects on*Macaranga harveyana*were within the uninfested site

There were fewer extrafloral nectar sources in the uninfested site. Within the infested site, 50% and 32% of the trees sampled along the two transects had EFNs, compared to 17% and 34% respectively in the uninfested site. These differences were significant only in the 2010 sample (χ^2^ = 7.5, df = 1, P = 0.013; and χ^2^ = 0.03, df = 1, P = 0.1 respectively for the two sample times) indicating that transect location greatly influenced the observations. Similarly, the occurrence of honeydew-producing insects differed greatly between the two sites, with 24% and 33% respectively (average of 29%) of assessable trees within the infested site harbouring these insects compared to only 7% and 0% within the two transects of the uninfested site. But these differences were statistically significant only in the October sample (χ^2^ = 7.04, df = 1, P = 0.013; and χ^2^ = 3.07, df = 1, P = 0.109 for the two sample times respectively). Although not formally quantified, the abundance of the insects was clearly different between the two sites, with those in the infested site predominantly occurring as clusters of many individuals, whereas only two individual scales were found within the uninfested site on two trees.

## Discussion

There is great variation in the effects that an invasive species will have throughout its exotic range, dependent upon the abiotic suitability of the habitat and the local co-existing biota [42], [43], [44].The impacts of *A. gracilipes* on Nu’utele varied greatly with season, but were consistent with knowledge of its impacts globally, and largely consistent for invasive ants generally. First, impacts are density dependent, with greatest negative effects occurring when invasive ant densities are highest. The serious negative consequences of this ant on land crabs is well documented from Christmas Island [25], but these crab deaths only occur at high ant densities (card counts greater than 38; Parks Australia North unpublished data) such as those found here during the May sample. Also, [45] found hermit crabs on Tokelau could not persist in areas where ant counts on individual cards exceeded 25. Invasive ant impacts on native ant communities are also dependent upon the density of the invasive ant, with greatest impacts where the invader has highest population densities [39], [46], [47].

Second, displacement of other ant species by *A. gracilipes* is relatively poor and appears to be limited to species of approximately equivalent size or larger. In northern Australia, 62% of species collected co-existed with *A. gracilipes* and the relative contribution of ants smaller than *A. gracilipes* to total abundance and species richness was always greater in infested sites [26]. All quantitative studies within the Seychelles have found many ant species smaller than *A. gracilipes* coexisting with the invader, but not the considerably larger species *O. simillimus* [40], [48], [49]. In Tokelau, all ant species coexisting with *A. gracilipes* are relatively smaller [22], [50], and in all other studies where species-specific data are not provided, ant diversity is either not, or only slightly, reduced in the presence of *A. gracilipes* [51], even on Christmas Island, where *A. gracilipes* attains the greatest reported ant densities in the world [21], [52]. Here, most other ant species were much smaller than *A. gracilipes,* with only *O. simillimus* being within the vulnerable size class. But despite the clear differences between the abundance of *O. simillimus* between the infested and uninfested areas in both the October (8 vs 100) and May samples (9 vs 200), this alone did not result in overall ant community differences.

Surprisingly, ant species richness may be greatest in places where *A. gracilipes* is present [40], [49]. This interesting observation, which is counter to most impact research for this and other invasive ant species, is likely to be because high-quality habitat for *A. gracilipes* is also likely to be high-quality for most co-occurring species, coupled with most ant species being smaller than *A. gracilipes*, and therefore apparently much less susceptible to it, as detailed above. The similarity of habitat quality for *A. gracilipes* and other ants is supported by the finding that the number of plants and honeydew-producing insects capable of producing a carbohydrate supply for ants were greatest where *A. gracilipes* was present, and where other ant species richness was also greatest. It is not possible to state whether the current distribution of *A. gracilipes* solely at the north-eastern end of Vini beach is a consequence of the vegetation composition, and hence carbohydrate availability, or if this distribution is merely by chance and in time the ant will infest the entire beach. Similarly, it is unclear whether the honeydew-producing insect density is a cause or consequence of the *A. gracilipes* distribution and abundances, or the presence of other ants, particularly the other exotic ant species. However, carbohydrate sources from both plants and honeydew-producing insects are well known drivers of invasive ant activity [12], [53], [54] and abundance [25], [55] just as their absence or poor quality is believed to be a clear limitation to invasions [34], [35], [41]. Further research into the links between carbohydrate supply and ant invasions is likely to yield great insights into the dynamics of ant invasions and their management.

Although little can be confirmed about the impacts of *A. gracilipes* on non-ant invertebrates from this spatially limited study, the consistent pattern of fewer spiders within the invaded site is consistent with expectations. Invasive ants overwhelmingly negatively impact other invertebrates, but such impacts are highly context specific [16], including for spiders [38], [56] and a predictive understanding of these dynamics, the mechanisms, as well as the ecological consequences, remain rudimentary. Although impacts would be expected for other invertebrates, especially given the high density of *A. gracilipes* found here, because of the small size of the infestation, and the mobility of most non-ant invertebrate groups, impacts would likely not be distinct until the infestation became considerably larger.

Male reproduction in October was consistent with findings from most places globally [27], [28], [29], [57] (Figure 1). However, such patterns were not consistent for queen reproduction (Figure 1). No queens were excavated in October, which was when queen reproduction was anticipated to occur, especially given that males were being produced. The only queen pupae that were collected were from a nest excavated in May, which is outside of the known reproductive period for this species in Australia and the Pacific [27], [28] and at the beginning of the dry season. However, *A. gracilipes* populations in India have been recorded to produce queens in May [58], and in the Seychelles sexuals can be produced throughout the year [29]. Reasoning for the great phenological variation throughout *A. gracilipes*’ range is unclear, but there are likely to be two interacting factors. First, the greatest reproductive driver is believed to be the onset of a wet season after an extended dry season [27], [58], and the regional timing and extent of this seasonality varies greatly. Second, *A. gracilipes* has an unusual, and as yet unresolved, reproductive strategy [59], [60], [61], which might also vary throughout its range. Regardless, of the drivers, these reproductive nuances are particularly noteworthy from a management perspective because best-practice treatments aiming to eradicate *A. gracilipes* have been found to be those that are conducted in times outside of the period of queen reproduction (B. Hoffmann, unpublished data). Clearly in Samoa, the reliance on biological knowledge simply obtained from other locations throughout its invasive range would result in an inappropriate treatment regime. Additional sampling in Samoa is required to determine the exact timing for production of sexuals.

The worker abundance levels found here during the time of high abundance were among the highest recorded in the world, notably as high as those seen on Christmas Island, but were lower than on Christmas Island during the time of low abundance. The abundance levels in Samoa were also similar but higher than those found in Arnhem Land, Australia (the only other location where card counts have been used), where card counts rarely exceed 38, and are on average only 17 [23]. Similarly, pupal abundance from Samoa in both sample periods was higher than those from Arnhem Land, Australia (B. Hoffmann, unpublished data). It appears likely that there is also a difference in the period of highest ant abundance in Samoa with pupal abundance increasing earlier in Samoa than in Arnhem Land (B. Hoffmann, unpublished data). The implications of the seasonality of worker abundance on treatment efficacy remain unstudied for any pest ant species, but clearly higher ant abundances are positively related to greater impacts [39], [46], [47], and therefore a greater need for management. However, knowledge of the seasonality of worker abundance and nest densities are particularly useful for assessing treatment efficacy, because only with this knowledge can the relative influences of treatment and seasonality be differentiated when treatments are applied to entire infestations.

Nest density on Nu’utele was also among the highest recorded throughout the world. In the Seychelles, maximum nest density have been recorded at one per 14.9 m^2^, none being underground [29]. In comparable rainforest habitat in Arnhem Land, *A. gracilipes* nest densities were one per 6.3 m^2^ (B. Hoffmann, unpublished data). In New Guinea coconut palm plantations, [62] found ephemeral nests in leaf litter could occur up to one per 2 m^2^. Finally, on Christmas island, [21] found nest entrance densities reached 10.5 per m^2^, however at this density these entrances would not constitute discrete nests. Indeed what constitutes a discrete nest within the high density populations on Christmas Island is not clear (B. Hoffmann, pers. obs.). This nest density information is particularly useful for management as baseline data to measure treatment efficacy, and also to justify the sampling intensity of post-treatment assessments, which would be best applied at greater than the pre-treatment nest density to maximize the likelihood of detection of any persistent nests.

The high abundance of *A. gracilipes* on the Aleipata islands is potentially of great concern, given the conservation significance of the islands, as well as the global reputation of this ant for its negative and often severe ecological impacts. As for any invasive ant species, should the impacts be determined to be great enough to consider management of the species, and management is considered to be feasible, the biological information presented here forms a solid basis upon which to determine knowledge-based treatment protocols and assessment benchmarks. The type of targeted, site-specific research such as that described here should be an integral part of any eradication program for invasive species.
